# Effect of Leflunomide on the Abnormal Expression of Lipid Rafts and F-Actin in B Lymphocytes from Patients with Systemic Lupus Erythematosus

**DOI:** 10.1155/2015/832916

**Published:** 2015-05-18

**Authors:** Guang Fu Dong, Xiao Zhang, De Ning He, Ling Li, Guang Feng Zhang

**Affiliations:** ^1^Department of Rheumatology and Immunology, Guangdong General Hospital, Guangdong Academy of Medical Sciences, Guangzhou 510080, China; ^2^Department of Rheumatology and Immunology, Pingxiang People's Hospital, Pingxiang, Jiangxi 337055, China

## Abstract

*Purposes*. To investigate the possible changes in B cell subsets and in B cell expression patterns of lipid rafts (LRs) and F-actin in patients with SLE and whether leflunomide treatment may have effect on these changes.* Methods*. The B cell subsets and LRs expression were determined by flow cytometry and confocal microscopy, and F-actin expression was examined by confocal microscopy.* Results*. CD27^+^IgD^+^ B cell subsets were significantly decreased while CD38^+^CD95^+^ B cell subsets increased in SLE patients. The LRs levels of B cells were remarkably increased and positively correlated with SLEDAI and anti-dsDNA titer in SLE patients. The expression level of LRs was significantly higher in CD38^+^ B cells than CD38^−^ B cells and negatively correlated with C3 levels. The increased expression of LRs was associated with reduced expression of F-actin in the B cells from active SLE patients. Furthermore, in vitro treatment of the cells with A771726 reduced the expression level of LRs, attenuated the overaggregation of LRs, and normalized the distribution of F-actin.* Conclusions*. There were abnormalities in B cell subsets and LRs and F-actin expression of B cell from SLE patients. Modulation of B cell expression of LRs and F-actin by LEF could be a potential therapeutic target for SLE.

## 1. Introduction

Systemic lupus erythematosus (SLE) is a systemic autoimmune disease that is known to be associated with polyclonal B cell hyperreactivity [1]. The pathogenesis of SLE remains not well understood. A combination of genetic factors, environmental influences, and sex hormones may contribute to the susceptibility to the disease [2]. While T cells have been considered to play a central role in the pathogenesis of SLE before, emerging evidences indicate that B cells may be a more important player than T cells in the pathogenesis of SLE [1, 3–5].

Lipid rafts (LRs), the specialized cholesterol- and glycosphingolipid-rich microdomains in the cellular membrane, have been reported to play an important role in the activation of B cells, including effects on B cell antigen receptor- (BCR-) initiated signal transduction, endocytosis of BCR-antigen complexes, loading of antigenic peptides onto MHC class II molecules, and receipt of helper signals via the CD40 receptor [6, 7]. Lipid rafts are also linked to the actin cytoskeleton which is important for lymphocyte antigen presenting [7].

Actin is a multifunctional protein that forms microfilaments presented either as a free monomer called G-actin or as a polymer microfilament called F-actin. Via polymerization and depolymerization, the actin cytoskeleton regulates a number of cellular functions. Dynamic regulation of F-actin cytoskeleton is critical to numerous physical cellular processes, including cell motility, cell division and cytokinesis, cell signaling, and the establishment and maintenance of cell junctions and cell shape. Many of these processes are mediated by extensive and intimate interactions of actin with cellular membranes. The actin cytoskeleton has also been reported to be involved in B cell activation by participating in the processing of the maturation of MHC class II molecules, internalization of BCR, and interaction between Fc*ε*RI and lipid raft components [8, 9].

Abnormal expression of lipid rafts and F-actin on T cells in SLE patients has been studied well [10–13]. The expression pattern of LRs and F-actin on B cells in SLE patients remains not quite clear [14].

Leflunomide (LEF) is one of the most important immunosuppressive drugs and has been extensively used in the therapy of rheumatoid arthritis in recent years. It also showed great success in treatment of SLE in both animal models and patients, though it was still not recommended as one choice of therapeutic drugs for SLE until now [15]. Following intestinal absorption, LEF is rapidly converted into its active metabolite A771726 that has been known to inhibit dihydroorotate-dehydrogenase (DHODH) and tyrosine kinases, leading to decreased proliferation of T and B cells [16]. However, the specific mechanism of how LEF regulates lymphocyte activation remained unclear. Whether LEF is involved in modulation of LRs and F-actin expression to exert its therapeutic effect on SLE is not fully understood. A better understanding about the role of LEF in SLE will help us in using it appropriately.

To clarify the changes in B cell subsets and LRs and F-action expression pattern on B cells and determine the possible role of LEF in these changes in the patients with SLE, we measured CD19, CD38, CD95, CD27, IgD, GM1, and F-actin expression levels and their patterns on B cells from SLE patients. Our results suggest that there were abnormalities at B cell subsets and expression of LRs and F-actin on B cells, which could be modified by A771726 in SLE.

## 2. Patients and Methods

### 2.1. Patients and Healthy Controls

54 SLE patients (51 females, 3 males; average age: 32.06 ± 11.38), 15 sex-matched (all females) primary Sjögren's syndrome (pSS) patients, and 20 age- and sex-matched healthy volunteers (19 females, 1 male; average age: 30.60 ± 10.78) who served as controls were enrolled for this study. The diagnosis of SLE was according to the 1997 revised American College of Rheumatology (ACR) criteria for the classification of SLE [17]. The diagnosis of pSS was according to the 2002 American-European Consensus Group criteria [18]. Disease activity in the SLE patients was assessed using the SLE Disease Activity Index (SLEDAI). In our cohort, SLE patients were divided into two subgroups based on SLEDAI including 37 active SLE (SLEDAI > 6) and 17 inactive SLE (SLEDAI ≤ 5) [19]. None of the patients were treated with statins which could alter lipid raft domains. All patients either were not exposed to or have discontinued for at least 2 weeks glucocorticoid and immunosuppressant drugs before recruitment into this study.

This study was performed according to the principles of the Declaration of Helsinki and each participant completed written informed consent before measurements. This study was also approved by the Local Ethics Committee (Institutional Review Board of Guangdong General Hospital).

### 2.2. Flow Cytometric Analysis for Peripheral Blood B Cell Subsets

15 mL EDTA-treated blood samples were obtained after an overnight fasting. Peripheral blood mononuclear cells (PBMCs) were isolated by density gradient centrifugation over Ficoll-Paque Plus. For immunofluorescence staining, the following monoclonal antibodies (mAbs) were used: CD5-PE (BD Pharmingen), CD19-FITC/CY5 (eBioscience), CD27-PE (eBioscience), CD38-CY7 (BioLegend), CD95-PE (eBioscience), and IgD-FITC (Invitrogen). To define B cell subpopulations, 10^6^ PBMCs were incubated with mAbs for 30 minutes on ice. The PBMCs were simultaneously stained with different fluorescein washed with PBS twice. Four-color immunofluorescence analysis was performed on the same day by flow cytometry (Bechman Coulter Inc.). The fetched cells suspension was gated for lymphocyte and at least 10,000 lymphocyte events were acquired per sample.

### 2.3. Isolation and Culture of B Lymphocytes

B cells were isolated from PBMCs by positive selection using MACS anti-CD19 microbeads and MS columns (Miltenyi Biotec, Gmbh, Germany) following the manufacturer's instructions. Flow cytometry consistently showed that the percentage of B cells in the isolated subpopulation was >90% as determined by anti-CD19 mAb staining. B lymphocytes were cultured in serum-free RPMI-1640 medium at 37°C for 4 hours, with or without A771726 (kindly provided by Cinkate Corporation, China) (at 0 *μ*g/mL, 15 *μ*g/mL, 30 *μ*g/mL, 45 *μ*g/mL, and 60 *μ*g/mL).

### 2.4. FACS Analysis of CTB Binding to the Plasma Membrane of B Cells

The binding of cholera toxin B subunit (CTB) (Sigma) is frequently used to identify lipid rafts, and the capacity for binding CTB is upregulated during B cell activation [20]. To analyze lipid raft, we stained cells with fluorescence-labeled CTB, which binds to the raft-associated glycosphingolipid GM1, previously shown to be a reliable marker for the detection of lipid raft domains [21]. In vitro A771726-treated B cells were fixed with 2% paraformaldehyde (PFA) at room temperature (RT) for 15 min. Fixed cells were resuspended in 100 *μ*L phosphate buffered saline (PBS) and then stained with CTB-FITC (final concentration 2 *μ*g/mL). At least 5000 cells were analyzed. To analyze surface CD38 or CD95 coexpression with lipid rafts in B cells, PBMCs were isolated and were stained with fluorochrome-conjugated mouse monoclonal anti-CD19, anti-CD38, and anti-CD95. Cells were fixed with 2% PFA at RT for 15 min and subsequently stained with CTB-Alexa Flour 488 (Molecular Probes Inc.) (final concentration: 10 *μ*g/mL). Four-color immunofluorescence analysis was performed.

### 2.5. Visualization of Lipid Rafts and F-Actin in B Cells by Confocal Microscopy

Ex vivo and in vitro A771726-treated B cells were washed by PBS and added to poly-L-lysine cover slips and mounted to slides. Cells were fixed with 2% PFA at RT for 15 min. Staining for F-actin was achieved with rhodamine phalloidin (Cytoskeleton Inc., Denver, USA) at RT for 30 min. Membrane staining for lipid rafts was achieved with CTB-FITC (final concentration: 10 *μ*g/mL) at 4°C for 30 min. Green and red fluorescence were recorded using a 40x objective. The colocalization of lipid rafts and F-actin was quantified using the “colocalization” tool in the Leica software.

### 2.6. Statistical Analyses

Data are expressed as median value and the rank. Differences between two groups were compared using the nonparametric Mann-Whiney test for unpaired data and Wilcoxon test for paired data. One-way ANOVA was used for comparison between more than two groups. Correlation was analyzed by Spearman's rank correlation test. *P* values less than 0.05 were considered significant.

## 3. Results

### 3.1. Abnormal B Cell Subsets in SLE

Initially, an analysis of CD5 expression in peripheral B cells was carried out in 20 active SLE patients and 11 healthy subjects. The result showed that the percentage of B cells in lymphocytes was significantly increased in SLE patients compared with normal subjects (median 16.7% (range: 2.5–48.0) versus 10.6% (range: 5.7–17.2)) (*P* = 0.01). But there was no difference in the frequency of CD5 expression on B cells between SLE patients (median 24.9% (range: 6.9–68.3)) and normal individuals (median 27.1% (range: 10.8–56.7)) (*P* = 0.409).

**Figure 1:**
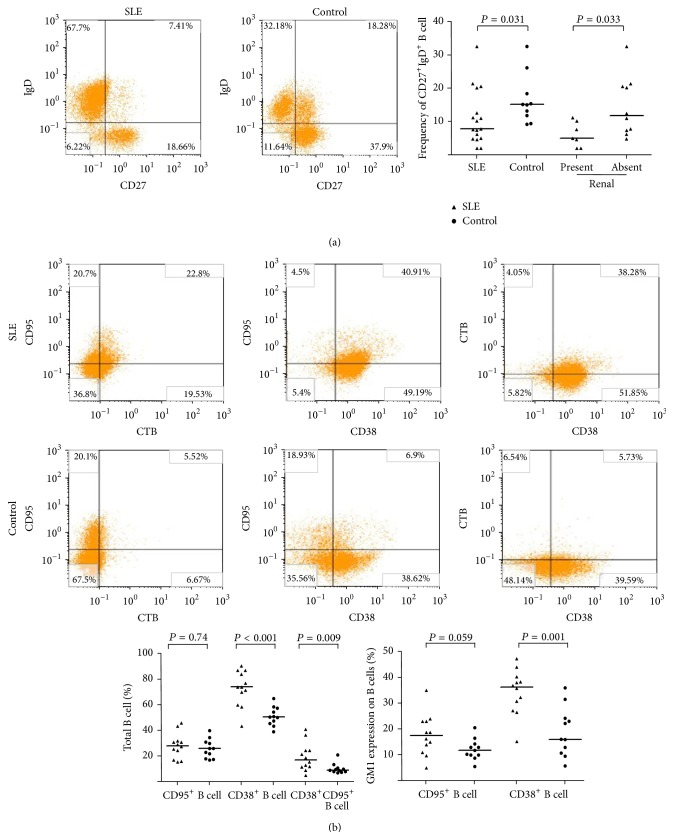
Surface phenotype of peripheral blood B cells was analyzed by* flow cytometry* and GM1 expression on B cells was detected by* confocal microscopy*. (a) SLE patients had a lower expression of CD27^+^IgD^+^ B cells than healthy controls, *P* < 0.05. SLE patients with renal injury had a higher expression of CD27^+^IgD^+^ B cells than SLE patients with no renal injury, *P* < 0.05. (b) The frequency of either CD38^+^CD95^+^ B cells or CD38^+^ B cells was significantly higher in SLE patients than in healthy controls, but there was no difference in the percentage of CD95^+^ B cells between SLE patients and healthy controls. The coexpression of CD38 and GM1 in SLE B cells was increased significantly in SLE patients. Horizontal line represents the median.

Further analysis of the expression of surface IgD and CD27 in B cell subsets in 17 active SLE patients and 10 healthy subjects showed no difference in CD27^+^ B cells and IgD^+^ B cells between SLE and normal control (CD27^+^ B cells: median 45.1% (range: 10.9–80.5) versus median 38.9% (range: 27.5–70.1), *P* = 0.941; IgD^+^ B cells: median 52.8% (range: 15.6–88.8) versus median 64.1% (range: 47.7–72.4), *P* = 0.711). However, a decrease in CD27^+^IgD^+^ B cell subset was observed in patients with SLE (median 7.7% (range: 2.0–32.6) versus 15.1% (range: 9.1–32.6)) (*P* = 0.031). The proportion of CD27^+^IgD^+^ B cell in patients with lupus nephritis (*n* = 10, median 5.0% (range: 2.0–11.1)) was lower than those in SLE patients without renal involvement (*n* = 7, median 11.9% (range: 4.7–32.6)) (*P* = 0.032) (Figure 1(a)). But there was no difference in the percentage of CD27^−^IgD^−^ B cells between SLE patients and normal control individuals.

Finally, we analyzed CD38 and CD95 expression in B cells surface. 12 active SLE patients and 11 healthy subjects were studied. There was no difference in the percentage of CD95^+^ B cells between SLE patients and normal controls, but an increased expression of CD38^+^ B cells was found and the percentage of CD38^+^CD95^+^ B cells was significantly increased (Figure 1(b)) in active SLE patients.

### 3.2. The Expression Level of CTB-Binding Lipid Rafts in SLE B Cells

**Figure 2:**
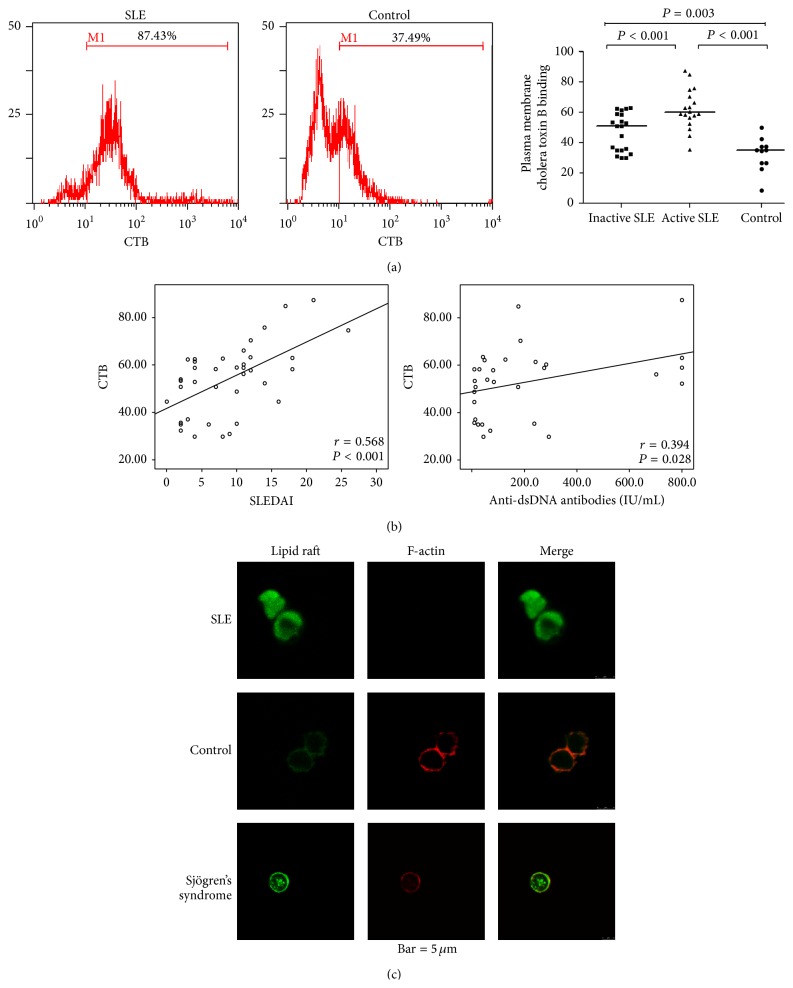
Altered expression of lipid rafts and F-actin in SLE B cells was analyzed by flow cytometry and confocal microscopy. (a) Expression of lipid rafts on SLE B cells was analyzed by flow cytometry. SLE B cells have more lipid rafts compartments than normal B cells. Active SLE B cells have more lipid rafts compartments than inactive SLE B cells. (b) The association between the percentage of lipid rafts (CTB) expressing B cells and the SLEDAI-score or anti-dsDNA antibody titer was analyzed by Spearman's rank correlation test. There was a significant positive correlation between the percentage of lipid rafts (CTB) expressing B cells and the SLEDAI-score or anti-dsDNA antibody titer. (c) Expression pattern of lipid rafts and F-actin in B cells was detected by confocal microscopy. There were different expression patterns of lipid rafts and F-actin in B cells from SLE patients, Sjögren's syndrome patients, and control subjects.

The LRs expression level in B cells was remarkably increased in SLE patients (median 57.0% (range: 29.7–87.4), *n* = 38) compared with those in healthy controls (median 34.8% (range: 8.4–50.0), *n* = 11) (*P* < 0.001). The LRs level on B cells was significantly higher in active SLE patients than in inactive SLE patients (median 59.6% (range: 35.3–87.4), *n* = 18, versus median 50.7% (range: 29.7–62.7), *n* = 20) (*P* < 0.001) (Figure 2(a)). In addition, the LRs level in B cells in inactive SLE patients was still higher than that in healthy controls (*P* = 0.003). There was a positive correlation between the LRs level in B cells and SLEDAI (*r* = 0.568, *P* < 0.001) (Figure 2(b)). Likewise, the LRs level in B cells showed a significant positive correlation with anti-dsDNA antibody titer (*r* = 0.394, *P* = 0.028). No correlation was seen between the LRs level in B cells and blood IgG level (*r* = −0.203, *P* = 0.291) or complement factor C3 level (*r* = −0.036, *P* = 0.843).

Analysis of CD38 and CD95 coexpression with LRs in PBMCs from 12 active SLE patients and 11 healthy controls revealed a significant increased LRs expression on CD38^+^ B cells in SLE patients compared with healthy controls (Figure 1(b)). Interestingly, in active SLE patients, LRs expression in CD38^+^ B cells (median 36.1% (range: 15.1–47.3)) was significantly higher than that in CD38^−^ B cells (median 10.5% (range: 4.1–31.7)), *P* < 0.001. The increased percentage of LRs expression in CD38^+^ B cell was correlated with complement factor C3 level (*r* = −0.718, *P* = 0.013).

### 3.3. Colocalization Pattern of F-Actin and Lipid Rafts in SLE B Cells

To analyze the colocalization of lipid rafts and F-actin, purified B cells from 7 active SLE patients, 6 pSS patients, and 4 healthy controls were doubly stained with CTB-FITC and rhodamine phalloidin. Confocal microscopy images revealed that, in B cells from healthy controls and pSS, LRs were small and uniformly distributed on the plasma membrane. F-actin was found mainly in a cortical distribution around the perimeter of the cell. This pattern was different from the pattern seen in B cells from SLE patients, which presented with stronger staining and an irregular large clustering of LRs companying with a decrease in F-actin level (Figure 2(c)).

### 3.4. Effects of A771726 on the Expression of Lipid Rafts and F-Actin on SLE B Cells

**Figure 3:**
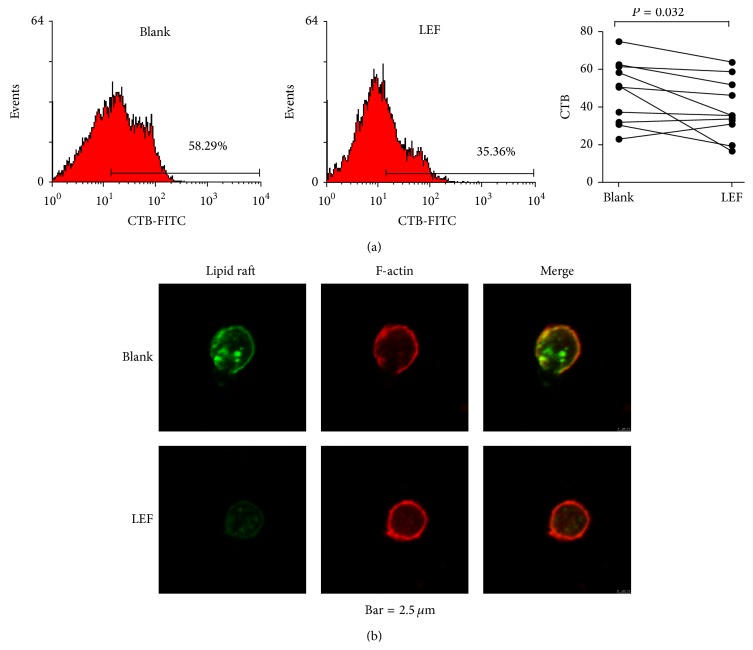
A771726 treatment altered the lipid rafts and F-actin expression in B cells in vitro. (a) Expression of lipid rafts on SLE B cells before and after A771726 treatment was analyzed by flow cytometry. A771726 treatment significantly reduced the lipid rafts expression level in SLE B cells. (b) Confocal microscopic profiles of B cells from 3 SLE patients show the modified lipid rafts and F-actin expression patterns after A771726 treatment. Magnification used was ×40 objective.

Our preliminary experiments found that A771726 did not obviously affect LRs level at the concentration of 15 or 30 *μ*g/mL. To investigate the effects of A771726 on LRs and F-actin on B cells, purified B cells from 10 active SLE patients were cultured with or without A771726 (45 *μ*g/mL or 60 *μ*g/mL) at 37°C for 4 hours. LRs were stained with CTB-FITC and analyzed by FACS. The percentage of CTB-binding LRs was significantly decreased after A771726 treatment and does not show significant difference between the concentration of 45 *μ*g/mL and the concentration of 60 *μ*g/mL (Figure 3(a)). Coexpression analysis of LRs and F-actin on SLE B cells by confocal microscopy showed that treatment of A771726 reduced the cluster of LRs associated with reversed distribution of F-actin (Figure 3(b)).

## 4. Discussion

Several studies have documented the specific perturbations of B cell maturation and differentiation in SLE and that alteration of B cell subsets and B cell phenotypes could play critical roles in SLE diseases. [1, 22], but the possible inherent B cell abnormalities in SLE patients remain to be defined clearly. Our results showed that there was an increased frequency of CD38^+^ B cells and decreased percentage of CD27^+^IgD^+^ B cells in SLE patients, but no difference of CD5^+^ B cells, CD27^+^ B cells, and IgD^+^ B cells between active SLE patients and normal controls was found. Moreover, SLE patients with lupus nephritis had lower level of CD27^+^IgD^+^ B cells than SLE patients without renal involvement. Previous studies had described a new population of memory B cells lacking expression of both CD27 and IgD, and increased frequency of this subset of B cells was associated with higher disease activity index [23]. In our study, we observed no significant difference in the percentage of CD27^−^IgD^−^ B cells between SLE patients and normal controls. However, our study showed that the percentages of CD38^+^ B cells and CD38^+^CD95^+^ B cells were significantly increased in active SLE patients, although there was no difference in the percentage of CD95^+^ B cells between SLE patients and normal controls. These different results may be attributed to SLE hyperheterogeneity, ethnic's heterogeneity, and grouping criteria heterogeneity. It is known that the increased expression of CD38^+^ B cells is closely associated with memory B cells hyperactivation and differentiation, while the increased expression of CD95 on the B cells may induce B cell apoptosis [24]. Further study is required to reveal the clinical relevance of the increased CD38^+^CD95^+^ B cells in the pathogenesis and progression of SLE.

Abnormal B cell activation is a prominent feature of the SLE [25]. Increased lipid raft expression on the plasma membrane of B cells may constitute a way to promote B cell activation [26]. In our study, we found that the lipid raft levels in B cells were dramatically increased in SLE patients, and the lipid raft levels in B cells were positively correlated with SLEDAI and dsDNA antibody titers. This suggests that the changes of lipid raft expression pattern on the B cells may contribute to B cell overactivation and disease activity in the SLE patients. Moreover, our study showed that the percentage of CD38^+^GM1^+^ B cells in SLE was significantly increased. CD38 is associated with the synthetic metabolism of B cells and also participates in B cell activation and proliferation through regulating gene transcription and protein phosphorylation. CD38 expression can also lead to increase of intracellular Ca^2+^ level and enhanced BCR signaling [27]. We speculated that the increased CD38 expression on B cells in SLE patients might decrease the threshold of B cell activation by promoting B cell lipid raft overexpression. The distinct expansion of the CD38^+^GM1^+^ B cells in SLE patients may be one of the major features of B cell inherent abnormality and related to SLE pathogenesis. Further mechanistic studies are required to reveal the relationship between CD38 and lipid rafts in B cells.

The actin cytoskeleton regulates lipid raft dynamics and coalescence in B cells. Depolymerization of the actin cytoskeleton could alter lipid rafts clustering and influence the threshold for cellular activation [28]. Whether lipid rafts and F-actin expression could be changed in B cells in SLE has not been reported so far. We found that lipid rafts were irregularly distributed on the plasma membrane and showed aggregation in SLE B cells, accompanied by a decrease in F-actin expression and abnormal distribution of F-actin, suggesting the abnormal expression of LRs and F-actin may contribute to the hyperactivation of B cells in SLE patients.

LEF is a potent effective disease-modifying antirheumatic drug and has been recently demonstrated to be safe and effective for the treatment of SLE and even refractory lupus and lupus nephritis [29, 30]. But it was still not recommended by the latest ACR/Eular treatment guidelines for SLE [31]. Our data showed that LEF treatment can reduce the lipid rafts levels and reverse the abnormal distribution of lipid rafts and F-actin of B cells in the active SLE patients. This finding suggests that regulating the abnormal expression of lipid rafts and F-actin in B cells may be a new mechanism for LEF to effectively treat SLE and that modulation of abnormalities of lipid rafts and F-actin expression on B cells could be an important new target in the SLE therapy.

## 5. Conclusions

Although this study was somewhat limited in statistical power because of the small sample size, our data showed that there were significant abnormalities in the B cell subsets and their expression pattern of lipid rafts and F-actin from SLE patients. Modifying these abnormalities may represent a new mechanism for LEF in effective treatment of SLE. The altered lipid rafts and F-actin expression pattern or their signaling pathways may be used as new targets in SLE treatment.

## Abbreviations

SLE: Systemic lupus erythematosus
pSS: Primary Sjögren's syndrome
LEF: Leflunomide
LRs: Lipid rafts
SLEDAI: SLE Disease Activity Index
CTB: Cholera toxin B subunit
dsDNA: Double-stranded deoxyribonucleic acid
A771726: The active metabolite of leflunomide.
